# Experimental Study on the Wind Erosion Resistance of Aeolian Sand Solidified by Microbially Induced Calcite Precipitation (MICP)

**DOI:** 10.3390/ma17061270

**Published:** 2024-03-09

**Authors:** Jing Qu, Gang Li, Bin Ma, Jia Liu, Jinli Zhang, Xing Liu, Yijia Zhang

**Affiliations:** 1 Shaanxi Key Laboratory of Safety and Durability of Concrete Structures, Xijing University, Xi’an 710123, China; 2 State Key Laboratory of Coastal and Offshore Engineering, Dalian University of Technology, Dalian 116024, China

**Keywords:** MICP, aeolian sand, wind erosion resistance, model test, solidified mechanism

## Abstract

Microbially induced calcite precipitation (MICP) is an emerging solidification method characterized by high economic efficiency, environmental friendliness, and durability. This study validated the reliability of the MICP sand solidification method by conducting a small-scale wind tunnel model test using aeolian sand solidified by MICP and analyzing the effects of wind velocity (7 m/s, 10 m/s, and 13 m/s), deflation angle (0°, 15°, 30°, and 45°), wind erosion cycle (1, 3, and 5), and other related factors on the mass loss rate of solidified aeolian sand. The microstructure of aeolian sand was constructed by performing mesoscopic and microscopic testing based on X-ray diffraction analysis (XRD), Fourier-transform infrared spectroscopy (FTIR), and scanning electron microscopy (SEM). According to the test results, the mass loss rate of solidified aeolian sand gradually increases with the increase in wind velocity, deflation angle, and wind erosion cycle. When the wind velocity was 13 m/s, the mass loss rate of the aeolian sand was only 63.6%, indicating that aeolian sand has excellent wind erosion resistance. CaCO_3_ crystals generated by MICP were mostly distributed on sand particle surfaces, in sand particle pores, and between sand particles to realize the covering, filling, and cementing effects.

## 1. Introduction

Arid and semi-arid regions account for 40% of the world’s total land area. Under the action of wind erosion, land desertification and sandstorm events have happened more frequently in these regions, further deteriorating the ecological environment. Therefore, it is urgent to implement wind prevention and sand solidification. Traditional sand fixation methods include mechanical sand fixation [1], plant sand fixation [2], and chemical sand fixation [3]. However, these methods have disadvantages such as a long construction period, high cost, and environmental pollution. The microbially induced calcite precipitation (MICP) technique is an emerging sand solidification method characterized by high efficiency, environmental protection, and durability [4]. With respect to the principle of MICP mineralization, the microorganisms that produce urease are used to hydrolyze urea into $CO_{3}^{2-}$, which generates CaCO_3_ deposits together with Ca^2+^ in the environment for solidification [5]. MICP has been applied to improve soils’ properties [6], crack repair [7], treatment of pollution soil [8], and waste [9]. Ghalandarzadeh et al. [10] used MICP to improve the unconfined compressive strength of kaolinite clay. Behzadipour et al. [11] explored the application of MICP technology in improving the shear strength of gold mine tailings. The experimental results showed that compared with the untreated samples, the cohesion intercept and friction angle of the treated tailings samples increased by about 19 kPa and 5°, respectively. Prongmanee et al. [12] stabilized clayey soil by producing ammonium carbonate supernatant to generate calcite precipitation. The results showed that the soil stabilized with calcite had higher compressive strength than the untreated soil. The microcosmic test showed that calcite filled the voids between soil particles and resulted in the denser package of soil. Wang et al. [13] studied the effect of temperature on the cemented structure of sand treated by MICP. The results showed that the generation of CaCO_3_ crystals by temperature would lead to changes in the internal friction angle, cohesion, stiffness, peak strength, residual strength, and expansion of sand samples treated by MICP. When carbonate crystals produced at 4 °C and 50 °C were fewer and smaller, they had lower strength reinforcement. In contrast, more larger crystal clusters were produced at 20 °C and 35 °C, which have effectively reinforced the sand particles. Jiang et al. [14] prepared cementing solution in hydrochloric acid solution to promote the solidification rate in the MICP reaction and evaluate its effectiveness. Research had shown that this method promoted the rapid bonding of calcareous sand particles, resulting in an unconfined compressive strength (UCS) of 1312.6 kPa for the sand column after five treatments. Compared with the conventional test group, the UCS of the test group containing HCl increased by about 1357%. Naskar et al. [15] investigated the effect of MICP on the mechanical properties of coal fly ash (CFA). After research, it was found that the specimens cured by MICP had higher strength, stiffness, and cohesion. Within a 28-day processing period, the permeability of CFA decreased by 78%, and the precipitation rate of calcite increased by 8%. Dubey et al. [16] conducted biological cementation treatment on desert sand through MICP and carried out soil erosion tests in indoor wind tunnels. The test results showed that single doses of 0.5 M and 1 M cementing solutions could continuously produce crusts with depths of 2 cm and 3.5 cm, thus effectively reducing erosion under the maximum velocity of 55 km/h. Devrani et al. [17] treated sand with MICP and biopolymer. Wind tunnel tests showed that the threshold friction velocity (TFV) increased from 20 km/h of the untreated sand to 45 km/h of the sand treated by MICP and biopolymer. They also observed that the mass loss rate of sand decreased from 75.23% of the untreated sand to 0%. Hang et al. [18] formed a cemented layer on the surface of desert sand through MICP to resist erosion and studied various factors relating to the erosion resistance of desert sand. The results showed that the erosion resistance of desert sand and the penetration resistance of sand surfaces were improved with the increase in treatment temperature and cementing solution concentration.

MICP technology has high bonding strength. It is not only suitable for soil reinforcement but also has a broad application prospect in controlling pollution elements, repairing cracks, repairing cultural relics, and anti-seepage treatment. However, aeolian sand has small particles, low water content, poor permeability, and low shear strength. Further validation is necessary for aeolian sand solidification using MICP. By taking aeolian sand solidified by MICP as the research object and conducting a small-scale wind tunnel model test, we analyzed the effects of wind velocity, deflation angle, wind erosion cycle, and other related factors on the mass loss rate of solidified aeolian sand and evaluated the erosion resistance of solidified aeolian sand. All the findings of this study provide an important reference value and scientific basis for the practice of wind prevention and sand solidification in desert regions.

## 2. Materials and Methods

### 2.1. Test Materials

**Figure 1:**
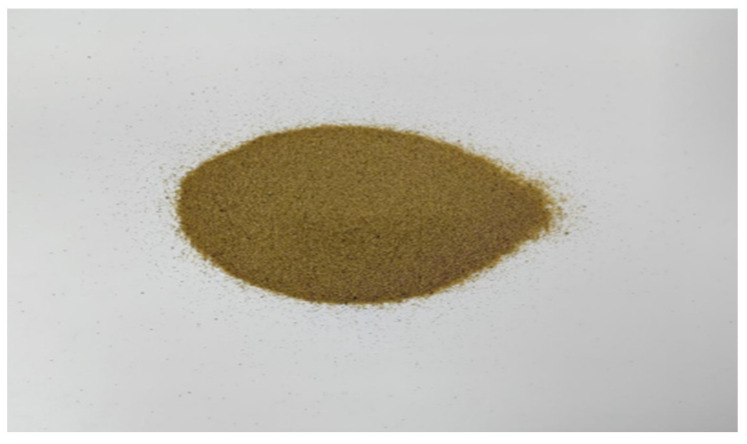
Aeolian sand taken from Mu Us Desert in Yulin, Shaanxi, China.

In order to reduce the negative effects of sandstorm brought by Mu Us Desert to the northwest of China, aeolian sand was taken from the desert (Figure 1). The physical properties of aeolian sand were tested in accordance with the standard for the geotechnical testing method (GB/T 50123-2019) [19]. The main physical properties of aeolian sand are shown in Table 1. Aeolian sand can be identified as poorly graded fine sand according to the coefficient of curvature and coefficient of nonuniformity.

The test strain was *Sporosarcina pasteurii* (ATCC 11859), purchased from Shanghai Bioresource Collection Center. The bacterial culture medium contained 5 g of NaCl, 20 g of agar powder, 900 mL of purified water, and 100 mL of 20% urea. During the test, the pH value of the culture solution was adjusted to 9.0, and the solution was sterilized under a high-pressure steam at 125 °C for 20 min. After the solution was cooled to 60 °C, 100 mL of sterilized 20% urea was added. Then, 1 mL of bacterial solution (OD_600_ = 1.0) was inoculated into the culture solution, and the mixed solution was placed onto a shaking table at 30 °C and 200 rpm for culture. The culture was stopped when the bacterial solution concentration of OD_600_ was 1.5, and the mixed solution was then placed into a 4 °C refrigerator for storage. The mixed solution of urea and calcium chloride with an equal concentration and volume was prepared as the cementing solution with a concentration of 1.25 mol/L and pH of 9.0.

### 2.2. Sample Preparation

**Figure 2:**
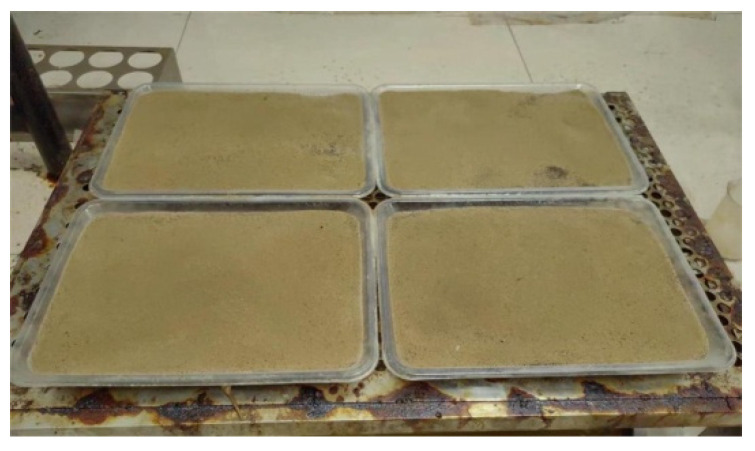
Sand table device.

During the test, an appropriate amount of aeolian sand was filtered by a 0.5 mm sieve and stored for further use. A small transparent plastic tray was used as a sand table for sample preparation. The top side of the sand table was 23.5 cm long, the bottom side was 15.4 cm long, the width was 16.5 cm, and the height was 3.0 cm. In order to ensure the discharge of bacterial and cementing liquid during the solidification process of the sample, 9 small holes with a diameter of 0.5 cm were drilled at the bottom of the sand tray at equal intervals. To prevent sand from accumulating at the bottom and flowing away, a layer of filter paper was laid at the bottom of the tray before loading. Subsequently, 40 sand tables were prepared under the condition of 1.45 g/cm^3^ dry density for the wind tunnel model test. Among them, 36 were treated with MICP curing and 4 were loose sand. The test set the loading height of aeolian sand at 2.5 cm and calculated that the sand required for each sand table was 1050.9 g, and that the volume was 724.78 cm^3^. Seventy milliliters of bacterial solution was evenly sprayed to the surface of aeolian sand using a handheld sprayer. Seventy milliliters of cementing solution with the same concentration was sprayed 1 h later. The steps above were repeated every 24 h to spray the bacterial solution and cementing solution 5 times, respectively. The wind tunnel test was conducted 10 to 15 days after the solidification samples were dried. The mass of the solidified aeolian sand samples was 1489.7 g (Figure 2).

### 2.3. Test Method

**Figure 3:**
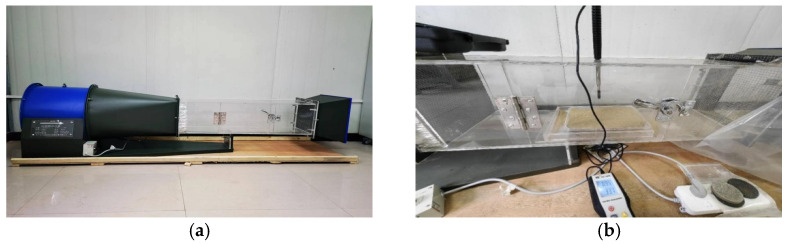
Wind tunnel testing equipment: (**a**) wind tunnel testing machine; (**b**) anemometer.

The wind tunnel test was carried out by means of the SNDY-4000 wind tunnel testing machine (manufactured by Nanjing Meiwen Science and Education Instrument Co., Ltd., Nanjing, China) (Figure 3a). The test section of the wind tunnel testing machine was 1.0 m long, 0.3 m wide, and 0.3 m high, with a wind rate of 11,000 m^3^/h and a rotating speed of 2800 r/min. During the test, the HT-9829 anemometer (manufactured by Shanghai Shouni Electric Technology Co., Ltd., Shanghai, China) was used to measure the wind velocity, and the anemometer was calibrated before each test to ensure the accuracy of the velocity, as shown in Figure 3b. Taking into account the effect of wind velocity (*v*), deflation angle (*α*), wind erosion cycle (*n*), and other related factors on the quality loss rate of aeolian sand (*φ*) considered during the test, we set 5 min as the deflation time. The next blow was carried out at an interval of 5 min, thus completing a cycle (Table 2). After the completion of all tests in each group, the mass loss rate of aeolian sand was obtained by measuring the mass of aeolian sand samples before and after the test. A lower mass loss rate of aeolian sand samples indicates stronger resistance of the samples against wind erosion.

The action mechanism of aeolian sand solidification by MICP was unveiled by selecting MICP solidified aeolian sand as samples, conducting XRD (BrukerAXS D8 X-ray diffractometer, Bruker Corporation, Billerica, MA, USA), FTIR (Thermo Scientific Nicolet iS20 Fourier-transform infrared spectrometer, Thermo Fisher Scientific, Waltham, MA, USA), and SEM (ZEISS Sigma 300 high-resolution scanning electron microscope, Carl ZEISS, Jena, Germany) testsand comprising and analyzing the crystal phase, characteristic functional group, and microstructure of aeolian sand before and after solidification.

## 3. Results and Discussion

### 3.1. Analysis of the Effect Caused by Wind Velocity

**Figure 4:**
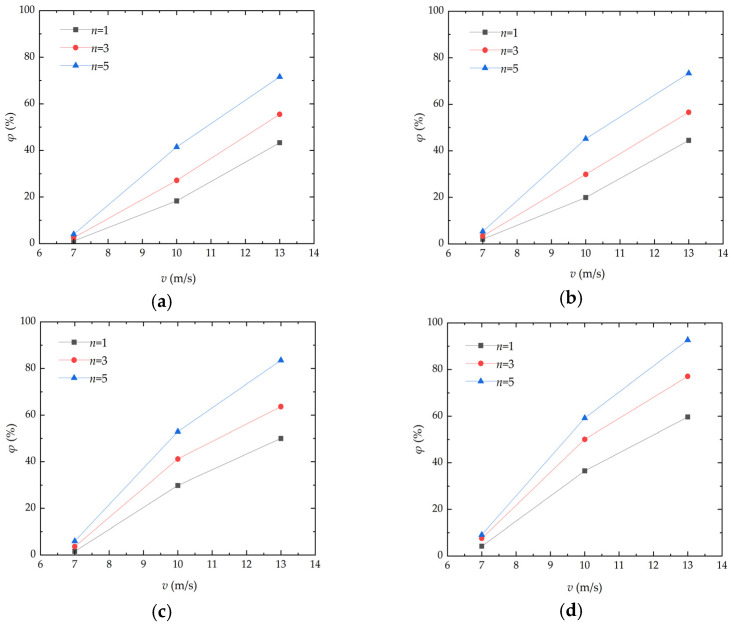
Curve of mass loss rate variation in aeolian sand with wind velocity: (**a**) *α* = 0°; (**b**) *α* = 15°; (**c**) *α* = 30°; (**d**) *α* = 45°.

Wind velocity has a significant effect on the threshold friction velocity (TFV), flying height, and migration distance of aeolian sand. Figure 4 presents the curve of mass loss rate variation in aeolian sand with wind velocity. As can be seen from the figure, the mass loss rate of aeolian sand is positively correlated with wind velocity and keeps increasing with the increase in the wind erosion cycle. When the deflation angle and number of wind erosion cycles are constant and the wind velocity increases from 7 m/s to 13 m/s, the maximum mass loss rate of aeolian sand reaches 92.68%. When the deflation angle is 15° and the number of wind erosion cycles is three, the mass loss rate of aeolian sand increases from 3.34% to 56.57% with the increase in wind velocity. When the deflation angle is 30° and the number of wind erosion cycles is fiveand the wind velocity increases from 10 m/s to 13 m/s, the mass loss rate of aeolian sand shows a downward trend compared with that in the wind velocity range of 7 m/s to 10 m/s. The fundamental reason for the above situation is that after cementation through MICP, a cemented layer with a certain thickness is formed on the surface of aeolian sand, thereby increasing TFV.A higher TFV usually indicates stronger resistance of samples against wind erosion [17]. The surface of aeolian sand is broken with the continuous increase in wind velocity. When the wind velocity reaches a certain level, the surface cemented layer is destroyed. As a result, the mass loss rate of aeolian sand in sand tables increases rapidly. After the loose aeolian sand is blown away, the mass loss rate tends to be stable. Nikseresht et al. [20] conducted similar wind tunnel tests. The samples were blown for 5 min at a wind velocity of 10 m/s, 20 m/s, and 30 m/s, and the soil loss was analyzed after repeated operation. The conclusion is similar to that in this paper, in that it can have a strong soil stabilization effect against wind erosion, which is primarily related to the formation of CaCO_3_ content. However, it is difficult to make a comprehensive comparison for different additives, wind speeds, and soil types.

### 3.2. Analysis of the Effect Caused by Deflation Angle

**Figure 5:**
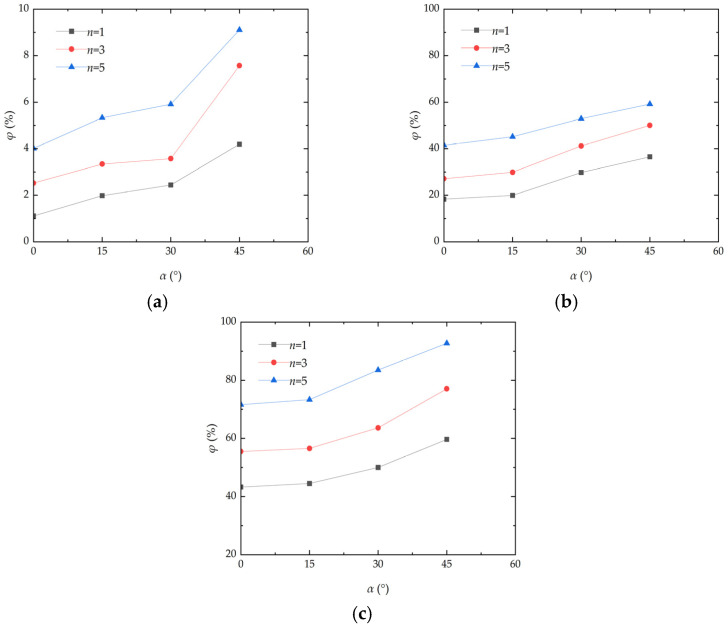
Curve of mass loss rate variation inaeolian sand with wind velocity: (**a**) *v* = 7 m/s; (**b**) *v* = 10 m/s; (**c**) *v* = 13 m/s.

Deflation angle has a significant effect on the mass loss rate of aeolian sand. Figure 5 presents the curve of the mass loss rate variation in aeolian sand with the deflation angle. As can be seen from the figure, the mass loss rate of aeolian sand increases with the increase in the deflation angle under different wind velocities. The mass loss rate of aeolian sand also increases with the increasing number of wind erosion cycles. When the wind velocity and number of wind erosion cycles are constant and the deflation angle increases from 0° to 45°, the mass loss rate of aeolian sand shows a stepwise upward trend. When the wind velocity is 13 m/s and the number of wind erosion cycles is one, the mass loss rate of aeolian sand increases from 43.28% to 44.46%, it then increases from 44.46% to 49.99%, and it finally increases from 49.99% to 59.66%. The fundamental reason for this is that with the continuous increase in the deflation angle, the wind erosion experienced by the samples increase and the corresponding TFV decreases, so that sand particles are blown away by wind more easily. As a result, the mass loss rate of aeolian sand changes steadily when the deflation angle increases from 0° to 30°, but it changes sharply when the deflation angle increases from 30° to 45°. Low wind velocity cannot destroy the solid surface of aeolian sand [21]. When the deflation angle increases, the surface area of wind acting on aeolian sand increases gradually, which will blow the solid sand lumps on the outer surface of aeolian sand away from sand tables, leading to a steady increase in the deflation mass of internal loose sand. Tominaga et al. [22] conducted a wind tunnel experiment of sand erosion/deposition around the cube. The conclusion is that as the wind velocity increased, the mass transport rate increased sharply. And, most importantly, considerable erosion occurred at the windward corners of the cube. All these findings can be echoed in this paper.

### 3.3. Analysis of the Effect Caused by Wind Erosion Cycles

**Figure 6:**
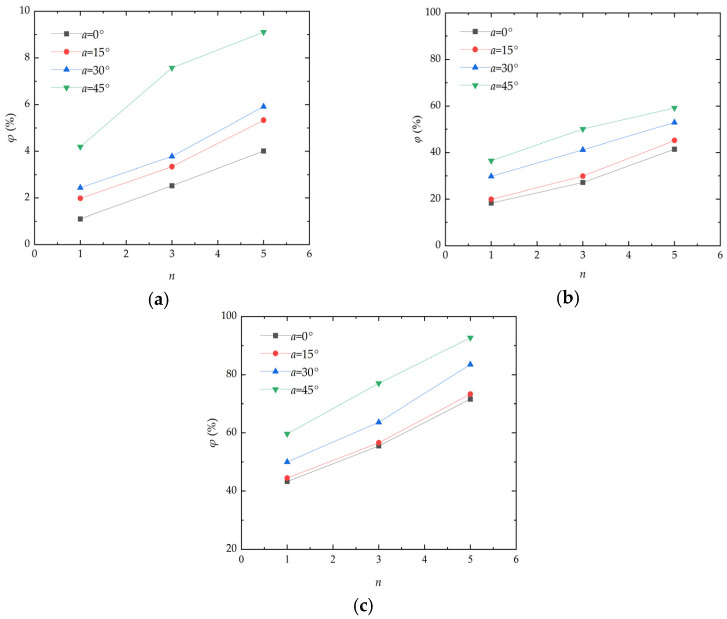
Curve of mass loss rate variation in aeolian sand with the number of wind erosion cycles: (**a**) *v* = 7 m/s; (**b**) *v* = 10 m/s; (**c**) *v* = 13 m/s.

The wind erosion cycle sfactor is one of the key factors affecting the mass loss rate of aeolian sand. Figure 6 presents the curve of the mass loss rate variation in aeolian sand with wind erosion cycles. As illustrated, under the action of different wind speeds, the mass loss rate of aeolian sand increases with the increase in wind erosion cycles, and the increase in amplitude becomes larger with the increase in the deflation angle. When the wind velocity is 7 m/s and the deflation angle is 45°, the mass loss rates of aeolian sand after 1, 3, and 5 wind erosion cycles are 4.19%, 7.57%, and 9.11%, respectively. When the wind velocity is 13 m/s and the deflation angle is 45°, the mass loss rates of aeolian sand after 1, 3, and 5 wind erosion cycles are 59.66%, 77.04%, and 92.68%, respectively. The fundamental reason for the above situation is that when there are fewer wind erosion cycles, the surface of aeolian sand only suffers weak erosion and the crust of aeolian sand is not completely destroyed, resulting in a lower mass loss rate of aeolian sand. When the number of wind erosion cycles increases, continuous wind erosion destroys the solidified layer of aeolian sand and the internal loosened aeolian sand will be further eroded, thereby increasing the mass loss rate of aeolian sand. The increase in the number of wind erosion cycles is equivalent to the increase in the total time of blowing erosion. Desert environments are harsh, and the ability of aeolian sand to withstand prolonged erosion is one of the major challenges.

### 3.4. Analysis of Morphological Characteristics of Aeolian Sand

**Figure 7:**
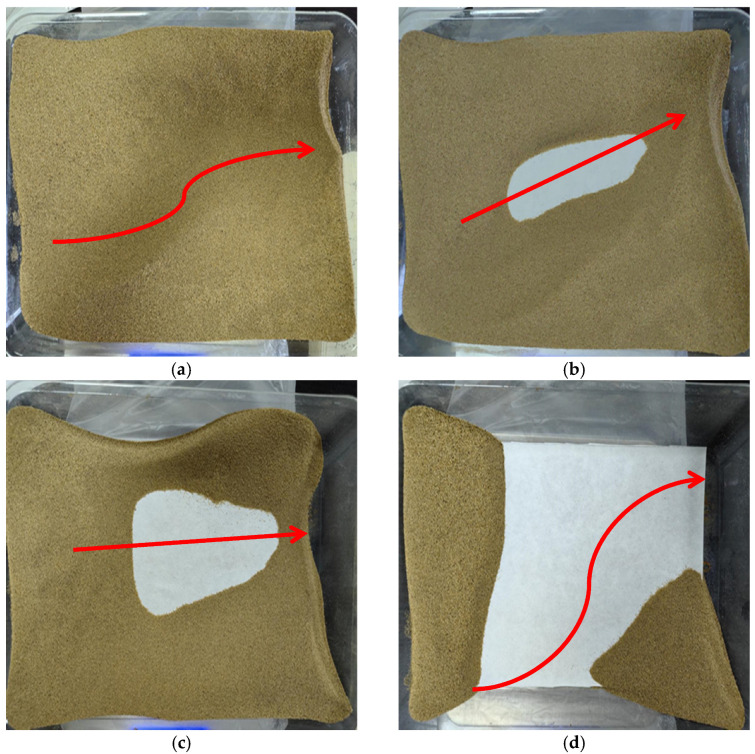
Wind erosion morphology of loose aeolian sand (The arrow indicate the dispersion direction of the aeolian sand): (**a**) *v* = 7 m/s, *α* = 15°, *n* = 1; (**b**) *v* = 10 m/s, *α* = 30°, *n* = 1; (**c**) *v* = 10 m/s, *α* = 30°, *n* = 3; (**d**) *v* = 13 m/s, *α* = 15°, *n* = 1.

Figure 7 presents the wind erosion morphology distributions of loose aeolian sand samples under different wind velocities, deflation angles, and wind erosion cycles; the deflation direction is from left to right. As can be seen from Figure 7a, when the wind velocity is 7 m/s, the deflation angle is 15°, and the number of wind erosion cycles is one; both the wind force and deflation angle are small, and the duration of wind action is short. Therefore, much aeolian sand is distributed in sand tables, and the aeolian sand moves from the middle to both sides. As can be seen from Figure 7b,c, the aeolian sand in the middle of the sand table is gradually blown away when the wind velocity is 10 m/s and the deflation angle is 30°, and the central blank area gradually increases with the increase in wind erosion cycles. According to Figure 7d, most of the aeolian sand in the sand table is blown and eroded, and only a small amount of aeolian sand is distributed at the end nearby wind source and the end far from the wind source when the wind velocity is 13 m/s, the deflation angle is 15°, and the number of wind erosion cycles is one. The above fact suggests that loose aeolian sand has poor resistance against wind erosion. Therefore, sandstorms and other natural disasters occur frequently in desert areas.

**Figure 8:**
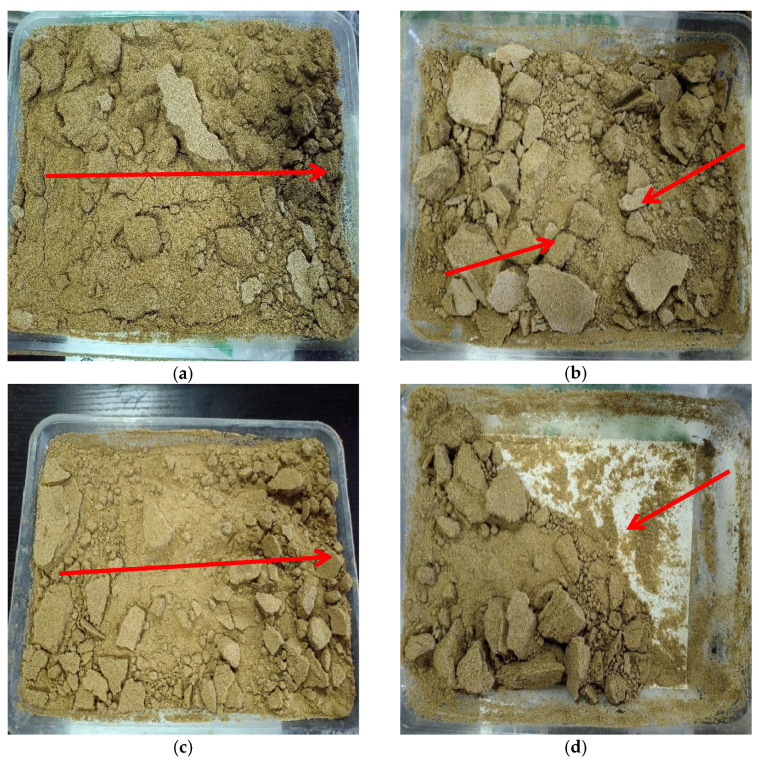
Wind erosion morphology of aeolian sand solidified by MICP (The arrow indicate the dispersion direction of the aeolian sand): (**a**) *v* = 7 m/s, *α* = 30°, *n* = 3; (**b**) *v* = 10 m/s, *α* = 30°, *n* = 3; (**c**) *v* = 10 m/s, *α* = 45°, *n* = 1; (**d**) *v* = 13 m/s, *α* = 30°, *n* = 3.

Figure 8 presents the wind erosion morphology distributions of solidified aeolian sand samples under different wind velocities, deflation angles, and wind erosion cycles; the deflation direction is from left to right. As figured, the deflation of solidified aeolian samples is significantly less than that of loose aeolian sand when the wind velocity is 7 m/s, the deflation angle is 30°, and the number of wind erosion cycles is three [23] because solid crusts can prevent aeolian sand from deflation to some extent. Small solidified aeolian sand samples are blown away when the wind velocity is 10 m/s, and some sand particles abrade sample surfaces during movement, which will further deteriorate the destruction caused by wind erosion. When the deflation angle is 45°, the area under the action of wind erosion increases, and the middle part of aeolian sand suffers more serious erosion than the two sides. We found that CaCO_3_ crystals mainly precipitated in the shallow layer because MICP only consolidated the surface layer of aeolian sand, and the experimental results of Wang et al. [24] are very consistent with this. When the wind velocity is 13 m/s, aeolian sand lumps are eroded and loose sand particles are blown away, resulting in a mass loss rate of 63.6%. Therefore, different from loose aeolian sand, a solid layer is formed on the surface of the aeolian sand solidified by MICP and has a certain ability to resist wind erosion. This will be of great help to reduce the occurrence of natural disasters in desert areas and reduce the current situation of air pollution.

### 3.5. Analysis of the MICP Solidification Mechanism

**Figure 9:**
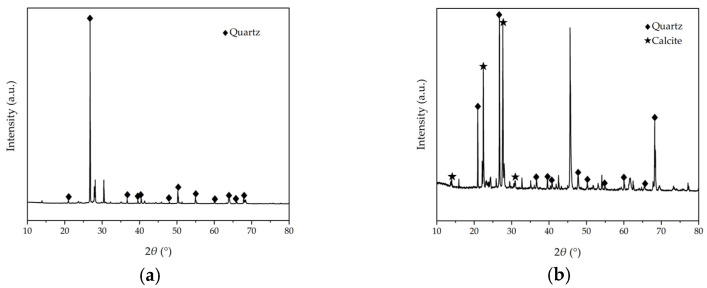
XRD spectrum: (**a**) loose aeolian sand; (**b**) MICP-solidified aeolian sand.

In order to compare the mineral composition of aeolian sand before and after solidification and evaluate the effect of the MICP solidification reaction on aeolian sand, the crystal phase of the samples was determined by carrying out an XRD test. Figure 9 presents the XRD spectrum of both loose aeolian sand and MICP-solidified aeolian sand. As shown, quartz is the main mineral component of loose aeolian sand, showing fewer diffraction peaks, but MICP-solidified aeolian sand shows more characteristic diffraction peaks. According to the analysis conducted using the software Jade 6 (TiLab, Beijing, China), they are the characteristic diffraction peaks of calcite, indicating that calcite is the main crystal settling in the samples treated by MICP.

**Figure 10:**
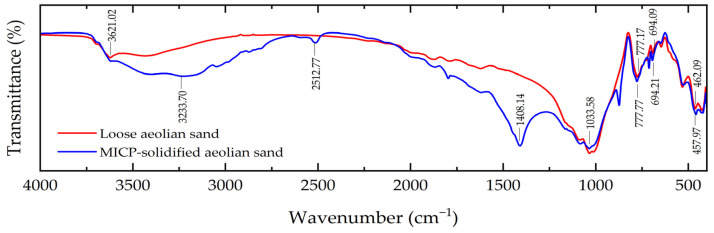
FTIR spectrum.

Figure 10 shows the FTIR testing results of both loose aeolian sand and MICP-solidified aeolian sand. As shown in the figure, the absorption peak of the two samples is mainly a narrow peak, and the position and quantity of absorption peaks vary slightly. The peaks at the location of 3621.02 cm^−1^ are O-H and N-H stretching vibration peaks; the peaks at the locations of 777.17 cm^−1^, 694.09 cm^−1^, and 462.09 cm^−1^ are Si-O symmetric stretching vibration peaks. The peak position at the location of 3233.70 cm^−1^ is the O-H stretching vibration peak, the peak at the location of 2512.77 cm^−1^ is the asymmetric vibration peak of $CO_{3}^{2-}$, and the absorption peak at the location of 1408.14 cm^−1^ is the C-O antisymmetric stretching vibration peak of $CO_{3}^{2-}$, as well as the characteristic peak of vaterite. The peak at the location of 1033.58 cm^−1^ is the C-O symmetric stretching vibration peak of $CO_{3}^{2-}$, indicating the presence of carbonate. The peaks at the locations of 777.77 cm^−1^, 694.21 cm^−1^, and 457.97 cm^−1^ are Si-O symmetric stretching vibration peaks, and SiO_2_ is the main component of sand particles, which is consistent with the XRD test results. It can be concluded that the carbonate generated in the process of MICP mineralization is CaCO_3_.

**Figure 11:**
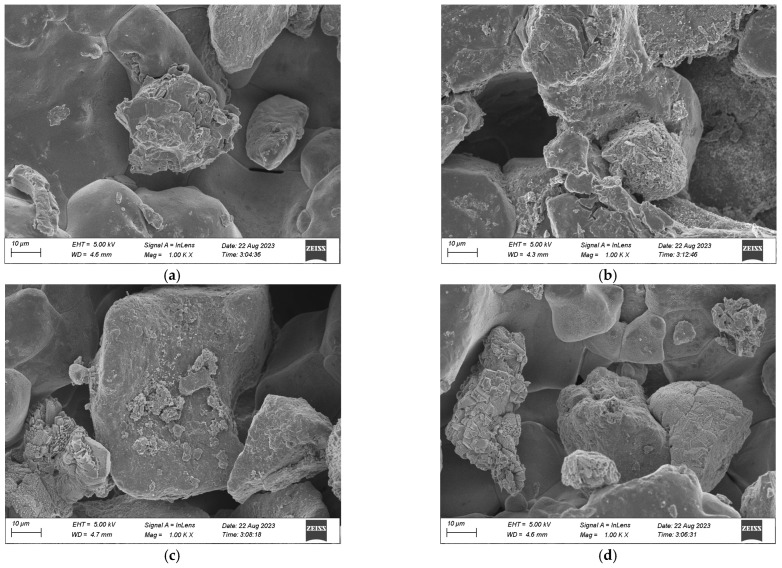
SEM image: (**a**) covering effect; (**b**) filling effect; (**c**) cementing effect; (**d**) calcite.

Figure 11 presents the microstructure of the aeolian sand solidified by MICP in order to further analyze the action mechanism of aeolian sand solidification by MICP. As illustrated, CaCO_3_ crystals generated by MICP mineralization are mostly distributed on sand particle surfaces, in sand particle pores, and between sand particles to achieve covering, filling, and cementing effects [25]. In the process of MICP mineralization, bacteria are first adsorbed on the surface of aeolian sand particles, providing nucleation sites for the formation and superposition of CaCO_3_ crystals [26]. The CaCO_3_ crystals deposit, accumulate, and grow between adjacent particles to cement the adjacent particles into a whole, turning the point contact between particles into surface contact and improving the overall stability of the samples. CaCO_3_ crystals can increase the surface roughness and cohesive force of sand particles, thereby improving the mechanical strength and erosion resistance of solidified aeolian sand. In Figure 11d, it can be seen that the CaCO_3_ crystals generated by MICP are diamond-shaped, indicating that the generated CaCO_3_ crystals are mainly calcite [27]. Calcite minerals are formed in the pores of particles to bind them. And there is a favorable relationship between its content and the strength of the sample [15]. These are consistent with the results of the XRD and FTIR experiments.

## 4. Conclusions

This study validated the reliability of the MICP sand solidification method by conducting a small-scale wind tunnel model test using aeolian sand solidified by MICP and analyzing the effects of wind velocity, deflation angle, wind erosion cycle, and other related factors on the mass loss rate of solidified aeolian sand. The microstructure of aeolian sand was constructed by performing mesoscopic and microscopic testing (XRD, FTIR, and SEM), thus revealing the mechanism of aeolian sand solidification via MICP. The main conclusions are as follows:

1. The mass loss rate of aeolian sand is positively correlated with wind velocity and keeps increasing with the increase in the wind erosion cycle. When the deflation angle and number of wind erosion cycle are constant and the wind velocity increases from 7 m/s to 13 m/s, the maximum mass loss rate of aeolian sand reaches 92.68%.
2. The mass loss rate of aeolian sand increases with the increase in the deflation angle. The mass loss rate of aeolian sand also increases with the increasing number of wind erosion cycles. When the wind velocity and number of wind erosion cycle are constant and the deflation angle increases from 0° to 45°, the mass loss rate of aeolian sand shows a stepwise upward trend.
3. Under the action of different wind speeds, the mass loss rate of aeolian sand increases with the increase in wind erosion cycles, and the increase amplitude becomes larger with the increase in the deflation angle. When the wind velocity is 7 m/s and the deflation angle is 45°, the mass loss rate of aeolian sand after 1, 3, and 5 wind erosion cycles is 4.19%, 7.57%, and 9.11%, respectively.
4. Loose aeolian sand has poor resistance against wind erosion. With the increase in wind velocity, the aeolian sand in the middle of the sand table is gradually blown away. Only a small amount of aeolian sand is distributed at the end near the wind source and the end far from the wind source. The solid layer formed on the surface of MICP-solidified aeolian sand has a certain ability to resist wind erosion. The mass loss rate of such aeolian sand is only 63.6% when the wind velocity is 13 m/s.
5. Quartz is the main mineral component of loose aeolian sand, while new calcite is the main mineral component of MICP-solidified aeolian sand. CaCO_3_ crystals generated by MICP mineralization were mostly distributed on sand particle surfaces, in sand particle pores, and between sand particles to achieve covering, filling, and cementing effects.
6. The solidification of aeolian sand will be particularly important for subgrade filling, soil anti-seepage, and erosion resistance, as well as slope protection. However, the environment in the desert area is complex and harsh, and factors such as freeze–thaw and ultraviolet light have not been effectively solved, which is expected to be supplemented and improved on in future related research.

## Figures and Tables

**Table 1:** Basic physical index of aeolian sand.

*G*_s_	*ρ*_dmax_ (g/cm^3^)	*ρ*_dmin_ (g/cm^3^)	*d*_10_ (mm)	*d*_30_ (mm)	*d*_60_ (mm)	*C*_u_	*C*_c_
2.65	1.85	1.47	0.086	0.120	0.198	2.30	0.85

**Table 2:** Wind tunnel testing scheme.

Deflation Angle (°)	Wind Velocity (m/s)	Wind Erosion Cycle
0	7	1, 3, 5
	10	1, 3, 5
	13	1, 3, 5
15	7	1, 3, 5
	10	1, 3, 5
	13	1, 3, 5
30	7	1, 3, 5
	10	1, 3, 5
	13	1, 3, 5
45	7	1, 3, 5
	10	1, 3, 5
	13	1, 3, 5

## Data Availability

Data are contained within the article.
